# Diet in Disease

**Published:** 1893-05-20

**Authors:** Ernest Hart

**Affiliations:** Bachelier-ès-Sciences-es-Lettres (rèstreint), formerly Student of the Faculty of Medicine of Paris, and of the London School of Medicine for Women


### Diet in Disease.

By Mrs. Ernest Hart, Bachelier-ès-Sciences-es-Lettres (rèstreint), formerly Student of the Faculty of Medicine of Paris, and of the London School of Medicine for Women.

### XXI.—UNDER-FEEDING AND OVER-EATING (*continued*).

The story is told of a great lady, who had been the wife in succession to three husbands, all of whom had been devoted to her—for she was a woman of unusual intelligence, beauty, and character—that she was once asked how it was that men so different in disposition as her three distinguished husbands had been so greatly attached to her, and by what secret charm she had chained their minds and their hearts. The great lady replied simply, "I feed them well." But it is said that the lady outlived all but her third husband, and the question which this story suggests is: "Are the well-fed the long-lived?" This involves another question: "What is the proper amount of food to take at different periods of life?"

**In Youth Food Should be Abundant.**—At this time the body is not only growing, but tissue change or metabolism is active, leading to vigorous life. The youthful body is, if healthy, intensely and restlessly active, and energy is redundant. Watch a family of children out walking with their governess or nurse; notice how they run, skip, and trundle their hoops, shout and laugh; filled to overflowing with the vigour and energy of life. This energy and the necessary growth of the body cannot be maintained without an abundant food supply. The food must also contain all the essential elements—albuminates to build up the muscular and other tissues, fats and starches to develop heat and energy, and mineral salts to aid in the healthy formation of bones and teeth. The diet may be as simple and wholesome as possible, the simpler the more wholesome; but there should be enough to eat to satisfy hunger. The greedy child is, as a rule, the ill-fed child; ill-fed in not having enough to eat, or in having food inappropriate to its age; for the modern custom of allowing children to partake of highly-flavoured dishes with their elders is as much to be deprecated as the starvation system which was in vogue at Dotheboys Hall. Meat, soups, milk, bread, butter, porridge, eggs, fruit, potatoes, green vegetables, farinaceous and sweet pudding, should be the staple articles of diet of growing boys and girls. Alcohol in any form is unnecessary and undesirable for young persons; even to the weak and delicate a cup of beef tea will be found to be more sustaining and stimulating than the "strengthening glass of port wine" which anæmic little girls are often persuaded to take. The anæmia of schoolgirls at the age of puberty is frequently caused by an insufficient meat dietary at school. Between the ages of twelve and sixteen, girls develop with great rapidity, both mentally and physically. The calls on their physical constitution are great, and can only be responded to by the body being well supplied with the materials out of which to manufacture energy and the elements of repair—in other words, by having girls well fed. It may be interesting and instructive to recall one's own experience of youth, and to record a dietary based on rigid principles, adopted and enforced to maintain health and to banish daintiness. One of a large family of children, I remember well the nursery and schoolroom dietary and regimen, to which all were submitted up to the age of fifteen. It was as follows: For breakfast, oatmeal porridge with milk and sugar, or bread and milk, on alternate days of the week, except on Sundays, when one boiled egg, and bread and butter was allowed. For midday dinner the fare was roast or boiled joint with potatoes and vegetables, and a sweet pudding or pie; for tea, bread, butter and jam. There was no restriction as to quantity, but what we took on our plates we were obliged to eat, it being looked upon as a disgraceful sign of greediness to take more than one could consume, or to ask for a second helping when appetite was satisfied. If we did so we were made to feel the discomfort of surfeit, a sure way of checking greedy demands for "more." As we lived in the country, ripe fruit and fresh milk were to be supplied *ad libitum.* Every day we were obliged to walk six miles along the roads and lanes, to go through half an hour's calisthenic exercises, and to have six hours' lessons. Riding on horseback, gardening, and playing filled up the rest of the time of a happy, healthy, and vigorous childhood. These personal reminiscences may be pardoned, as they illustrate my point that the dietary of children should be plain and abundant.

**In Adult Life.**—When the processes of digestion and assimilation are active, when all the organs are healthy and the body has the power of eliminating and discharging effete products, the intake of food may be in excess of the actual needs of the body without harm. This is true, however, only so long as active muscular exercise is taken, or great demands are made on the energy of the whole system. Englishmen are said to be the greatest meat eaters in the world, and they carry their carnivorous habits to whatever part of the world they inhabit, whether it be the tropics of India or the wintry plains of Canada; but they are at the same time the greatest athletes in the world, and the people of the most devouring and restless energy. The youth of England expend much of their strength and energy in walking, boating, cricket, tennis, and football, and if they did not do so they would soon become—if they continued the same diet—a dull, phlegmatic, and stupid race. The generous diet of adolescence, even of those who undertake active muscular exercise, must, however, be watched with care. The recurrent bilious attack, the frequent headache or *migraine*, or an increasing deposit of fat, show that the supply of food is greater than the demands of the body, and must be decreased if health is to be maintained. "I can accustom my body to ring alarums for food whenever I choose," said the wise Locke, and the regular recurrence of appetite at certain intervals and hours is no certain sign in such an automatic organ as the stomach, that food is absolutely required by the body. In a very few days a healthy person can easily accustom himself to get hungry at any hour of the day he chooses, or which is convenient for meals. If, however, an abundant dietary is dangerous, unless carefully watched by those who take daily active muscular exercise, it is more than dangerous, it is disastrous, to those who lead sedentary lives, or who are brain workers. The great majority of our adult middle-class population in cities lead sedentary lives, and it may be said unhesitatingly that they, as a rule, consume far too much albuminous food, butcher's meat in particular. The albuminoids of the food being not fully oxidised in the body by muscular exercise, remain as effete products, and ultimately give rise to dyspepsia, liver complaints, gout, and Bright's disease. One deplorable result of excessive meat eating in England is the ill-temper which is a chronic moral complaint among us. In no country, I believe, is home rendered so unhappy and life made so miserable by the ill-temper of those who are obliged to live together as in England. To everybody who reads these lines examples will occur of homes which are rendered quite unnecessarily unhappy, when they might be happy, by the moroseness and rudeness of the head of the family, or the peevishness of the wife, or the quarrelling of the younger members. If we compare domestic life and manners in England with those of other countries where meat does not form such an integral article of diet, a notable improvement will be remarked. In less meat-eating France, urbanity is the rule of the home; in fish and rice-eating Japan harsh words are unknown, and an exquisite politeness to one another prevails even among the children who play together in the streets. In Japan I never heard rude angry words spoken by any but Englishmen. I am strongly of the opinion that the ill-temper of the English is caused in a great measure by a too abundant meat dietary combined with a sedentary life. The half oxidised products of albumen form urates and uric acid, which, circulating in the blood, produce both mental and moral disturbances.

Brain-workers should live sparingly if they would work well and live long. Their force is required for mental exertion, and should not be expended on the task of digestion, for "they should remember that the digestion of heavy meals involves a greater expenditure of nerve force." Besides fish, eggs, milk, and light porous well-made bread, fresh vegetables and fruit should form their chief sustenance. They should take only a small amount of butcher's meat, and that especially at those times when they are able to take more physical exercise. Some animal fat is, however, useful, such as fresh butter or cream, or a rasher or two of fat bacon at breakfast. (Burney Yeo.) Women, whose bodies are smaller and whose energy is less, require as a rule less food than men, but the same strict dietetic rules cannot be adopted by them as by men, for during menstruation, pregnancy, and lactation, demands are made on their physical and nervous systems which can only be met by a more abundant food supply specially rich in albuminoids. Their diet must, therefore, be regulated more or less by the varying circumstances of their physical condition. By women, however, who lead sedentary lives—and they are the great majority—it must be remembered that a diet in which meat, eggs, and milk form a large part is not conducive to health, while, on the other hand, if obesity is to be avoided, farinaceous and saccharine foods must be taken in due proportion. The healthful thing to do is to lead an active and unselfish life, on a moderate diet, sufficient to maintain strength and not increase weight.

